# From Maxwell's equations to the theory of current‐source density analysis

**DOI:** 10.1111/ejn.13534

**Published:** 2017-03-28

**Authors:** Sergey L. Gratiy, Geir Halnes, Daniel Denman, Michael J. Hawrylycz, Christof Koch, Gaute T. Einevoll, Costas A. Anastassiou

**Affiliations:** ^1^ Allen Institute for Brain ScienceSeattleWA98109USA; ^2^ Faculty of Science and TechnologyNorwegian University of Life SciencesAasNorway; ^3^ Department of PhysicsUniversity of OsloOsloNorway; ^4^ Department of NeurologyUniversity of British ColumbiaVancouverBCCanada

**Keywords:** current transfer, electrical conductivity, electrical stimulation, extracellular recordings, field potentials

## Abstract

Despite the widespread use of current‐source density (CSD) analysis of extracellular potential recordings in the brain, the physical mechanisms responsible for the generation of the signal are still debated. While the extracellular potential is thought to be exclusively generated by the transmembrane currents, recent studies suggest that extracellular diffusive, advective and displacement currents—traditionally neglected—may also contribute considerably toward extracellular potential recordings. Here, we first justify the application of the electro‐quasistatic approximation of Maxwell's equations to describe the electromagnetic field of physiological origin. Subsequently, we perform spatial averaging of currents in neural tissue to arrive at the notion of the CSD and derive an equation relating it to the extracellular potential. We show that, in general, the extracellular potential is determined by the CSD of membrane currents as well as the gradients of the putative extracellular diffusion current. The diffusion current can contribute significantly to the extracellular potential at frequencies less than a few Hertz; in which case it must be subtracted to obtain correct CSD estimates. We also show that the advective and displacement currents in the extracellular space are negligible for physiological frequencies while, within cellular membrane, displacement current contributes toward the CSD as a capacitive current. Taken together, these findings elucidate the relationship between electric currents and the extracellular potential in brain tissue and form the necessary foundation for the analysis of extracellular recordings.

## Introduction

Electrical activity of excitable brain cells is realized by the transmembrane ionic currents which, in turn, give rise to currents and the corresponding scalar electric potential in the extracellular space. Measurements of extracellular potential therefore provide information about electrical activity in the brain and aid to unravel the function of the underlying neuronal circuits. The high‐frequency component (above ~500 Hz) of the extracellular potential, termed multi‐unit activity, is typically used to detect spiking of individual neurons (Schmidt, 1984). In contrast, the signal at lower frequencies (below ~200 Hz), termed the local field potential (LFP) (Buzsáki *et al*., 2012; Einevoll *et al*., 2013), characterizes the collective electrical activity of neuronal populations. At a spatial scale greater than that of a single cell, this collective electrical activity may be described by a spatially smooth three‐dimensional current‐source distribution, termed current‐source density (CSD) (Mitzdorf, 1985).

The idea that the CSD may be estimated from the Laplacian of the extracellular potential recorded at nearby locations within the brain, originates from Pitts (1952) and forms the basis of CSD analysis. Nicholson (1973) provided a theoretical justification for Pitts' insight in the special case of the quasi‐stationary approximation of Maxwell's equations (Haus & Melcher, 1989), which neglects both magnetic induction and displacement current. This theory tacitly assumes that tissue conductivity is independent of the frequency of the signal in the physiological range, and that diffusion, advection and displacement currents in the extracellular space are negligible in comparison to Ohmic drift current.

The validity of these assumptions, however, has been questioned in recent studies. Bédard & Destexhe (2009) developed a theoretical model predicting that ionic diffusion in the extracellular space is the main cause for the frequency dependence of the LFP. In a follow‐up study, it was concluded that the CSD must be due to the extracellular diffusion current rather than the transmembrane currents (Bédard & Destexhe, 2011). Furthermore, analyzing the extracellular potential recordings, Riera *et al*. (2012) found that the estimated laminar CSD profiles do not sum to zero across the cortical depth as would be expected from their neuronal origin. To address this paradox, they speculated that tissue polarization as well as diffusive and advective currents might need to be accounted for in the CSD analysis of extracellular potential recordings.

The limiting assumptions of the theory of CSD analysis (Nicholson, 1973; Nicholson & Freeman, 1975) and challenges to its validity from both experimentalists and theoreticians motivated us to revisit the physical basis of CSD analysis and examine its underlying assumptions. Starting with Maxwell's equations of macroscopic electromagnetism, we utilize the electro‐quasistatic approximation to establish the equations describing fields of physiological origin. We present the general relationship between currents and the potential in the extracellular space and motivate a coarse‐grained description needed for the analysis of electrophysiological recordings. Applying spatial averaging to currents in brain tissue, we arrive at the notion of the CSD of transmembrane currents and subsequently derive the equation for CSD analysis considering the possible frequency dependence of tissue conductivity. We show that, in general, the extracellular potential is determined by the transmembrane currents as well as by the gradients of the putative extracellular diffusive currents, which can play an important role at the lowest frequencies. In turn, the effect of the displacement and advective currents in the extracellular space is negligible as a result of fast charge relaxation. However, within cells the displacement current contributes toward the CSD as a capacitive current.

## Materials and methods

### Electrophysiological recordings

All surgeries and procedures were approved by the Allen Institute for Brain Science Institutional Animal Care and Use Committee. Recordings were made in C57BL/6 male mice, >12 weeks old (Jackson Laboratories, *n* = 2). Detailed descriptions of the experimental apparatus and procedures are available in a previously published report (Denman *et al*., 2016).

Briefly, an initial surgery was made to attach a headpost to the skull. Following surgery, the animal was allowed to recover for at least 7 days before habituation. Prior to recording, animals were allowed to fully habituate to head‐fixation in the experimental apparatus over several sessions of increasing duration. The apparatus consisted of a horizontal disk suspended in a spherical environment onto which light was projected. Animals were allowed to run freely on the disk while head‐fixed.

On the day of recording, anesthesia was induced and maintained with inhaled isoflurane (5% induction, 2–3% maintenance). A small craniotomy was made over primary visual cortex using stereotactic coordinates and a reference screw was implanted as far from the recording site as possible, rostrally, within the area of exposed skull. The animal was transferred to the experimental apparatus and allowed to recover from anesthesia. A high‐density array of extracellular electrodes, containing electrodes spaced every 20 μm vertically (Lopez *et al*., 2016), was lowered through the craniotomy; the dura matter was pierced by the electrode array. The array insertion continued until some electrodes were below the cortex and within underlying structures. At this level, several electrodes remained above the pial surface, ensuring complete coverage of cortex. After reaching this insertion depth, the electrode was allowed to rest untouched for at least 30 min before data were recorded.

Visually activity was evoked in cortex using brief full‐field luminance changes. Luminance changes were 50 ms in duration and alternated between increases and decreases in luminance, returning to a mean luminance (~3 cd/m^2^) for 3 s between changes. The magnitude of luminance changes was 0.2 cd/m^2^ for OFF and 5.8 cd/m^2^ for ON. Signals were acquired in two parallel data streams at 10‐bit resolution: a MUA data stream high‐pass filtered at 500 Hz and sampled at 30 kHz and a LFP data stream low‐pass filtered at 300 Hz and sampled at 2.5 kHz. The analyses presented were performed on the LFP data stream.

### Estimation of the CSD

The array data were mapped to the cortical depth locations after identifying the channel corresponding to the pial surface by visual inspection of raw LFPs post hoc. Brief (~500 ms) chunks of raw data from each channel were plotted in an arrangement that allowed comparison of neighboring channels; the channel at which amplitude dropped discontinuously and higher frequency components became more homogenous was chosen as the pial surface.

The CSD was estimated from the trial‐averaged cortical LFP recordings for both ON (*n* = 50) and OFF (*n* = 50) luminance conditions. To estimate the CSD we used a variant of the delta‐source iCSD method (Pettersen *et al*., 2006) assuming a radius of 0.5 mm for the circularly symmetric sources around the recording electrode. This method utilizes the solution of the Poisson equation, Eq. (22), for the extracellular potential *Φ*_*i*_ at the *i*‐th cortical location. It can be expressed as a linear superposition $\Phi_{i}=A_{ij}s_{j}$ of sources *s*_*j*_ at each of *j*‐th location, where *A*_*ij*_ is a forward operator. Correspondingly, the CSD may be estimated as ${\hat{s}}_{j}=W_{ji}\Phi_{i}$, where *W*_*ji*_ is the regularized inverse of the forward operator, which suppresses the contribution of the noise on the estimated sources (Gratiy *et al*., 2011). The tissue conductivity was taken at 0.3 mS/mm (Wagner *et al*., 2014).

The divergence of the diffusive current in Eq. (21) when expressed in terms of the ionic concentrations, is given by $-\nabla \cdot {\left\langle J^{\mathrm{dif}}\right\rangle }_{e}=F\sum_{i}z_{i}\nabla \cdot \left(D_{i}\nabla {\left\langle c_{i}\right\rangle }_{e}\right),$ where ${\left\langle c_{i}\right\rangle }_{e}$ is the coarse‐grained extracellular concentration of the *i*‐th ionic species. Assuming K+ and Na+ ions dominate the changes in the ionic concentration and utilizing the condition of electroneutrality $\left(\Delta {\left[K+\right]}_{e}+\Delta {\left[\text{Na}+\right]}_{e}=0\right)$, we find $-\nabla \cdot {\left\langle J^{\mathrm{dif}}\right\rangle }_{e}=F\left(D_{K+}-D_{\mathrm{Na}+}\right)\nabla^{2}{\left[K+\right]}_{e}$, which constitutes a Poisson equation for [K+]_*e*_. Therefore, the divergence of the diffusive current (i.e., the apparent CSD resulting from diffusion) may be estimated from measurement of [K+]_*e*_, applying the same technique as for estimating the CSD from the LFP recordings. Similarly, we assume that the diffusion current is localized to the same cylindrical volume as the CSD and varies only along the cortical depth. We use *D*_K+_ = 1.96 · 10^−9^ m^2^/s and *D*_Na+_ = 1.33 · 10^−9^ m^2^/s (Grodzinsky, 2011).

## Results

### Equations of electromagnetisms of physiological origin

Our starting point is the set of macroscopic Maxwell's equations describing electro‐magnetic field variables, which are spatially averaged over volumes that are large compared to atomic volumes (Russakoff, 1970; Griffiths, 2012):

(1) $$\nabla \times E=-\frac{\partial }{\partial t}B,$$

(2) $$\nabla \times H=J+\frac{\partial }{\partial t}D,$$

(3) ∇·D=ρ,

(4) ∇·B=0,

where *ρ* and ***J*** are the free (i.e., unbound) charge density and current density, ***E*** and ***B*** are the electric and magnetic fields, respectively. The effects of bound charges and currents are included in the auxiliary ***D*** and ***H*** fields, which may be expressed in terms of the fundamental ***E*** and ***B*** fields using constitutive relations. For linear materials with instantaneous response properties, it holds that ***D*** = *ɛ**E*** and $H=\frac{1}{\mu }B$ where *ɛ* is the electric permittivity and *μ* the magnetic permeability of the medium.

Spatial averaging over volumes including many atoms eliminates references to individual atoms and removes the high spatial frequency components of the field variables. Correspondingly, the macroscopic description may be viewed as a description for which the spatial Fourier component of the field variables above some limiting frequency *ξ*_lim_ are irrelevant and eliminated by performing averaging over volumes with the dimension ~1/*ξ*_lim_. The irrelevant spatial frequencies are determined not by the physical structure of the system, but rather by the particular problem we are attempting to solve (Robinson, 1971). As such, the macroscopic equations for a particular system may be formulated using different averaging volumes—all depending on the spatial scales relevant for the application to a particular problem.

Maxwell's equations describe a host of electromagnetic phenomena occurring across a wide range of spatial and temporal scales and are difficult to analyze in a general form. To describe the electric fields in the brain, we introduce two approximations which drastically simplify the mathematical treatment of electrodynamics.

Firstly, for fields of physiological origin, the typical temporal frequencies are so low (less than a few thousand Hz) that the magnetic induction $\frac{\partial }{\partial t}B$ has a negligible effect on the electric field (Plonsey & Heppner, 1967; Rosenfalck, 1969). The error in the electric field *E*_err_ at angular frequency *ω* relative to the actual field *E* made by neglecting the magnetic induction is given by $E_{\mathrm{err}}/E∼{\left(\omega \tau_{\mathrm{em}}\right)}^{2}$ (Haus & Melcher, 1989). Here, *τ*_em_ = *l*/*v* is the time it takes the electromagnetic wave to propagate across the characteristic length *l* at velocity $v=c/\sqrt{\mu_{r}\varepsilon_{r}}$ in a material having relative permittivity *ɛ*_*r*_ and permeability *μ*_*r*_, where *c* is the speed of light in vacuum. For example, in grey matter we may take the characteristic length ~1 mm, corresponding to the cortical thickness. Using measured values of permittivity and permeability in mammalian grey matter (e.g., Wagner *et al*., 2014), yields the relative error $E_{\mathrm{err}}/E<10^{-7}$ for frequencies in a range of 10 Hz to 10 kHz, so that magnetic induction can be safely neglected. Neglecting the magnetic induction in Faraday's law, Eq. (1), constitutes the electro‐quasistatic approximation (Haus & Melcher, 1989):

(5) ∇×E≈0⇒E=−∇Φ,

that is, the electric field is essentially conservative and can be expressed as a gradient of a scalar potential Φ. Consequently, using the electro‐quasistatic approximation to describe fields in the brain tissue of physiological origin amounts to a negligible error when compared to the exact solution using a full set of Maxwell's equations. In contrast, the displacement current $\frac{\partial }{\partial t}D$ in Ampere–Maxwell's law (Eq. (2)) is responsible for the capacitive charging of neural membranes and cannot be neglected.

Secondly, the macroscopic velocity ***u*** of ions in the brain and the magnetic field of physiological origin are so low that the magnetic component of the Lorentz force $F=q\left(E+u\times B\right)$ is negligible. Indeed, using the largest bulk flow velocity *u* ~ 1 m/s due to the arterial blood flow (Bishop *et al*., 1986), the typical magnetic field *B* ~ 100 fT (Hämäläinen *et al*., 1993) and extracellular electric field *E* ~ 1 V/m (Cordingley & Somjen, 1978) arising from neuronal activity, yields *uB*/*E* ~ 10^−13^. Consequently, the effect of the magnetic field of physiological origin on the motion of free charges is negligible in comparison to the effect of the electric field.

The negligibility of magnetic induction and magnetic component of the Lorentz force results in the decoupling of the electric and magnetic fields. As the current density is now independent of the magnetic field, it is convenient to eliminate the ***H*** field from consideration by taking the divergence of Eq. (2), resulting in a current continuity statement:

(6) $$\nabla \cdot J^{\mathrm{tot}}=0,$$

where the total current density $J^{\mathrm{tot}}\overset{\mathrm{def}}{=}J+\frac{\partial }{\partial t}D$ is solenoidal, that is, current travels along closed loops. Current continuity, Eq. (6), also represents the principle of charge conservation, which may be cast in a familiar form $\nabla \cdot J+\frac{\partial }{\partial t}\rho =0$ by expressing the displacement current in terms of the density of free charges *ρ* using Gauss's law, Eq. (3). Together with the constitutive relations, Eqs. (3), (5) and (6) determine the electric field, current density and charge density. Then, if desired, the magnetic field can be determined from the known current density by using Eqs. (2) and (4).

### Fine‐grained description of electric currents in the extracellular space

The extracellular space occupies ~20% of brain tissue volume and has a torturous geometry with a typical thickness of ~40–60 nm (Syková & Nicholson, 2008). It contains the interstitial fluid, which constitutes a dilute solution of mobile ions as well as the extracellular matrix, which is composed of a mesh‐work of long‐chain macromolecules including fixed charges. To resolve the electric field within the narrow confines of the extracellular space, we must select the linear dimension of the averaging volume to be shorter than the thickness of the extracellular space. On the other hand, here we will not be concerned with the details of the electric field on the spatial scale of the Debye length ~1 nm (Syková & Nicholson, 2008) characterizing the extent of electrostatic forces around individual charges (Grodzinsky, 2011). Choosing the size of the averaging volume with dimension ~10 nm allows both resolving the fields across the extracellular space as well as averaging out the strong electrostatic forces present at the shorter spatial scale. The chosen spatial scale is much finer than the dimensions of dendritic diameters (~1 μm). Thus, for the purposes of describing fields and currents in brain tissue, we will refer to it as a fine‐grained scale. Here, we present such a description and then motivate an alternative description at the coarser spatial scale needed for the analysis of the multi‐electrode LFP recordings.

Typically, the extracellular space is treated as a volume conductor by considering only the electromigration current arising in the presence of the electric field. However, more generally, the migration of ions in the interstitial fluid may also occur even in the absence of an electric field due to diffusion or advection (Probstein, 2005). The role of the extracellular diffusion current on the extracellular potential is debated and has been the subject of recent theoretical (Bédard & Destexhe, 2009, 2011) and modeling (Pods *et al*., 2013; Pods, 2017; Halnes *et al*., 2016) studies. In turn, the significance of advective mass transport within the bulk of the interstitial fluid is discussed in Abbott (2004), and was suggested to play a role for the CSD analysis (Riera *et al*., 2012).

The electromigration of ions is described by Ohmic drift ***J*** ^ohm^ = *σ**E***, where *σ* is the electrical conductivity. The diffusion current density of ions in a dilute solution $J^{\mathrm{dif}}=-F\sum_{i}z_{i}D_{i}\nabla c_{i}$ is driven by the gradients of ionic concentrations *c*_*i*_, where *D*_*i*_ and *z*_*i*_ are the diffusion coefficient and valence of the *i*‐th ionic species, respectively, and *F* is the Faraday constant. The advection current $J^{\mathrm{adv}}=uF\sum_{i}z_{i}c_{i}$ results from charge transfer within bulk flow in the interstitial fluid with velocity ***u***. Substituting the specific expressions for each current mechanism into Eq. (6) we find:

(7) $$\nabla \cdot \left(\sigma E-F\sum_{i}D_{i}z_{i}\nabla c_{i}+uF\sum_{i}z_{i}c_{i}+\frac{\partial }{\partial t}\left(\varepsilon E\right)\right)=0,$$

where we utilized the constitutive relation ***D*** = *ɛ**E***.

For physiological conditions in the extracellular space some of the mechanisms contributing to the total current may be neglected, which results in drastic simplification of Eq. (7). To compare the importance of the different current mechanisms, we express the electric field in Eq. (7) via the charge density using Gauss’ law, ∇·E=ρ/ε, and find:

(8) $$\frac{\rho }{\tau_{e}}+\nabla \cdot \left(-F\sum_{i}D_{i}z_{i}\nabla c_{i}+uF\sum_{i}z_{i}c_{i}\right)+\frac{\partial }{\partial t}\rho =0,$$

where for simplicity we neglect the possible inhomogeneity of conductivity and permittivity in the extracellular space and define the relaxation time constant *τ*_*e*_ = *ɛ*/*σ*. Charge relaxation is controlled by the mobile ions in the interstitial fluid. Consequently, the relaxation time constant is determined by the electrical properties of the interstitial fluid. The interstitial fluid has typically been assumed to possess similar composition to the cerebrospinal fluid (Syková & Nicholson, 2008) and correspondingly similar electrical properties. Using measured values of conductivity *σ* ~ 1.8 S/m (Baumann *et al*., 1997) and permittivity *ɛ* ∼ 9.6 · 10^−10^ F/m (Andreuccetti *et al*., 1997) in the cerebrospinal fluid at physiological frequencies, leads to *τ*_*e*_ ~ 10^−9^ s.

The charge density in the extracellular space may be expressed as a sum *ρ* = *ρ* ^*f*^ + *ρ* ^*m*^, where *ρ* ^*f*^ is the charge density of fixed charges in the extracellular matrix and *ρ* ^*m*^ = $F\sum_{i}z_{i}c_{i}$ is the charge density of mobile ions in the interstitial fluid. Accounting for the incompressibility of the interstitial fluid, ∇·***u*** = 0, and that the density of fixed charges does not change with time $\frac{\partial }{\partial t}\rho^{f}=0$, Eq. (8) becomes:

(9) $$\left(\rho^{f}+\rho^{m}\right)-\tau_{e}F\sum_{i}z_{i}\nabla \cdot \left(D_{i}\nabla c_{i}\right)+\tau_{e}\frac{d}{dt}\rho^{m}=0,$$

where $\frac{d}{dt}\rho^{m}\overset{\mathrm{def}}{=}u\cdot \nabla \rho^{m}+\frac{\partial }{\partial t}\rho^{m}$ is the derivative with respect to the moving fluid element (material derivative). For fields of physiological origin, we find that *ωτ*_*e*_ ≪ 1, and so the term $\tau_{e}\frac{d}{dt}\rho^{m}$ attributed to the contributions of displacement and advection currents is negligible in comparison to the term *ρ* ^*f*^ + *ρ* ^*m*^ attributed to the Ohmic current. Correspondingly, neglecting the advection and displacement components in Eq. (7) and utilizing the electro‐quasistatic approximation, E=−∇Φ , leads to the Poisson equation:

(10) $$\nabla \cdot \left(\sigma \nabla \Phi \right)=-F\sum_{i}z_{i}\nabla \cdot \left(D_{i}\nabla c_{i}\right),$$

where the source term on the right‐hand side arises from diffusion fluxes.

Notably, derivation of Eq. (10) does not require assuming electroneutrality. In fact, invoking electroneutrality would have resulted in a contradiction between Eq. (10) and Gauss’ law Eq. (3), which is, however, avoided when accounting for a non‐zero charge density (see Appendix B).

If the ionic concentrations are known, then the extracellular potential may be found from Eq. (10), that is, the solution of the forward problem, given the distribution of membrane currents along the boundary of the extracellular space. More generally, the concentration of ionic species would need to be determined from the solution of the Nernst–Planck equation (Probstein, 2005) simultaneously with the solution of Eq. (10).

However, Eq. (10) does not provide a practical way for interpreting the extracellular, multi‐electrode recordings in terms of neuronal currents, that is, solving the inverse problem, because it is severely underdetermined. The spatial resolution of extracellular recordings is limited by the distance between recording sites (typically ≳20 μm) along modern multi‐channel probes (Shobe *et al*., 2015; Lopez *et al*., 2016) and is too sparse to infer the detailed distribution of the boundary currents along the cellular membrane. The information about neuronal currents, which may be inferred from such data would be similarly limited in spatial resolution and could only represent some average measure over volume elements including multiple neurites. Thus, to analyze extracellular multi‐electrode recordings, it is necessary to develop the description of the extracellular potential in terms of currents in brain tissue at a much coarser spatial scale comparable to the resolution of experimental recordings. We will refer to it as a coarse‐grained scale.

### Coarse‐grained description of currents in brain tissue

As discussed in the section ‘Equations of electromagnetisms of physiological origin’, the macroscopic Maxwell's equations describe field variables which are spatially averaged over the macroscopic volume elements to eliminate the unwanted high spatial frequencies. At the coarse‐grained scale, the size of the averaging volume is chosen large enough to include components of multiple neurites, and thus would average over both neurites and the extracellular space, that is, over the neural tissue. Then, the corresponding macroscopic field variables would characterize the tissue properties and could not be used for the description of the extracellular space. To avoid blurring the distinctions between the two spaces, we use the fine‐grained macroscopic field variable, and then perform the second averaging (i.e., coarse‐graining) separately over the cellular and the extracellular space.

**Figure 1:**
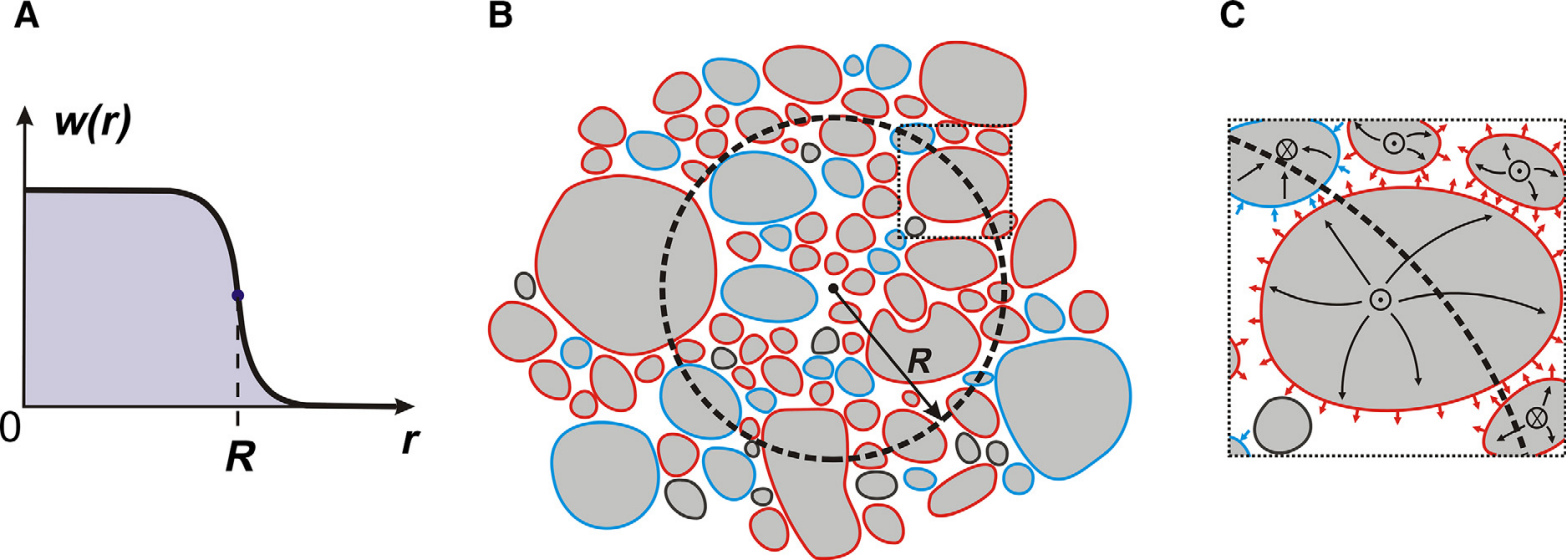
Spatial averaging of currents in brain tissue. (A) An example of a kernel *w*(*r*), which may be used in the spatial averaging procedure. The width of the plateau *R* is much larger than the size of the dendritic diameter. The function transitions to zero monotonically in a well‐behaved fashion to avoid jitter in the averaged variables. (B) Schematic of a cross‐section of neural tissue with neuropil (grey) surrounded by the extracellular space (white). Spherical averaging volume (shown by a dashed black circle) with an effective radius *R* corresponding to the width of the averaging kernel encloses multiple processes of several nearby cells. The CSD at the central location (black dot) is computed by summing membrane currents over the spherical averaging volume. The CSD computed over this volume may generally result from a combination of outward (red outline) and inward (blue outline) membrane currents. (C) Schematic of cellular current from a dotted rectangular detail in right‐hand corner in panel (B). The axial currents along the neuropil are shown as crosses or dots corresponding to the flow into or out of the paper, respectively. Some of the cytoplasmic current diverts toward the membrane (black arrows) and results in the outward (red arrows) or inward (blue arrows) transmembrane current. According to Eq. (15), the divergence of the averaged currents in the cellular space (black lines) may be found as a weighted sum of the transmembrane currents *J*_*m*_.

We define the coarse‐grained total current density:

(11) $$\left\langle J^{\mathrm{tot}}\left(r\right)\right\rangle =\int dv^{\prime }w\left(r-r^{\prime }\right)J^{\mathrm{tot}}\left(r^{\prime }\right)$$

as a convolution over the entire space with the averaging kernel *w*(***r***) being a real, non‐negative and continuous function normalized to unity: ∫ *dv*′*w*(***r***′) = 1. For the coarse‐grained current density to represent a smooth local average over multiple neurites, *w*(***r***) must vary slowly over the dimension of dendritic diameter (*d* ~ 1 μm) and approach zero in some well‐behaved fashion as shown in Fig. 1 A. Correspondingly, we demand the width of the kernel's plateau, that is, the effective radius *R* of the averaging spherical volume, to be much larger than the size of dendritic diameter: *R* ≫ *d*.

To distinguish between the currents in cellular (including neurons, glia and vasculature) and extracellular space (including interstitial fluid and extracellular matrix), we formally express Eq. (11) as a sum:

(12) $$\left\langle J^{\mathrm{tot}}\left(r\right)\right\rangle ={\left\langle J^{\mathrm{tot}}\left(r\right)\right\rangle }_{c}+{\left\langle J^{\mathrm{tot}}\left(r\right)\right\rangle }_{e}$$

of the averaged cellular ${\left\langle J\left(r\right)\right\rangle }_{c}=\int_{V_{c}}dv{}^{\prime }w\left(r-r^{\prime }\right)J\left(r^{\prime }\right)$ and extracellular ${\left\langle J\left(r\right)\right\rangle }_{e}=\int_{V_{e}}dv^{\prime }w\left(r-r^{\prime }\right)J\left(r^{\prime }\right)$ current densities, where the integration is performed only over the corresponding cellular *V*_*c*_ and extracellular *V*_*e*_ volumes, respectively, as shown in Fig. 1 B.

Both cellular and extracellular coarse‐grained current densities are defined over the whole tissue space, rather than only within their corresponding spaces. Therefore, the coarse‐graining procedure effectively introduces the bi‐domain representation, in which brain tissue is viewed as consisting of two interpenetrating cellular and extracellular domains with the corresponding two sets of field variables. A similar bi‐domain representation is widely used in the modeling of cardiac tissue and has successfully described the human electrocardiogram (Geselowitz & Miller, 1983; Henriquez, 1992). Such bi‐domain models describe the coarse‐grained extracellular and intracellular potential, which are coupled through the cable equation. In contrast, here, we present the formalism for describing the coarse‐grained extracellular potential in relationship with currents in brain tissue as motivated by the method of CSD analysis (Mitzdorf, 1985).

Averaging to the current continuity, Eq. (6), and utilizing commutativity between the differentiations and averaging operations over the entire space (see Appendix A) yields:

(13) $$\nabla \cdot \langle J^{\mathrm{tot}}\left(r\right)\rangle =0,$$

which is the statement of current continuity at the coarse‐grained scale. Substituting Eq. (12) into Eq. (13) results in

(14) $$\nabla \cdot {\left\langle J^{\mathrm{tot}}\left(r\right)\right\rangle }_{c}+\nabla \cdot {\left\langle J^{\mathrm{tot}}\left(r\right)\right\rangle }_{e}=0,$$

stating that the divergences of the coarse‐grained cellular and extracellular currents sum to zero.

The divergence of the coarse‐grained current density in the cellular domain may be expressed via a sum of transmembrane currents (see Appendix A)

(15) $$-\nabla \cdot {\left\langle J^{\mathrm{tot}}\left(r\right)\right\rangle }_{c}=\int_{S_{c}}da^{\prime }J_{m}\left(r^{\prime }\right)w\left(r-r^{\prime }\right),$$

weighted by the averaging kernel, where *J*_*m*_ is the transmembrane current density, and *S*_*c*_ is the surface of the cellular membrane. As membrane currents in the surface integral in Eq. (15) are weighted by the averaging kernel, the sum of membrane currents effectively includes contributions only within a vicinity around ***r***, where the kernel is non‐negligible. The benefit of Eq. (15) is in that it allows us to express the confounding cellular currents averaged over the cellular cytoplasm and membrane in terms of the weighted sum of the transmembrane currents as shown in Fig. 1 C. Because the averaging kernel is normalized, the integral on the right‐hand side of Eq. (15) has the units of current per unit volume and may be used to define the transmembrane current‐source density (CSD):

(16) $$s^{\mathrm{mem}}\left(r\right)\overset{\mathrm{def}}{=}\int_{S_{c}}da^{\prime }J_{m}\left(r^{\prime }\right)w\left(r-r^{\prime }\right),$$

which represents a continuous and smooth measure of electric current in and out of the extracellular space. The smoothness of the CSD is determined by the choice of the averaging kernel, which should be selected to achieve the desired spatial resolution for the description of the currents in brain tissue. The membrane current *J*_*m*_ in Eq. (16) is a sum of capacitive current $C_{m}\frac{\partial V_{m}}{\partial t}$ and ionic currents *J*_ion_, where *C*_*m*_ is the specific membrane capacitance and *V*_*m*_ is the transmembrane voltage. The capacitive current is in fact the manifestation of the displacement current within cellular membranes, while the ionic current is a sum of diffusive, advective and drift currents.

With such a definition of the CSD, Eq. (14) yields:

(17) $$\nabla \cdot {\left\langle J^{\mathrm{tot}}\left(r\right)\right\rangle }_{e}=s^{\mathrm{mem}}\left(r\right),$$

which links the coarse‐grained extracellular currents to the transmembrane CSD. Derived from charge conservation, Eq. (17) is in turn a statement of charge conservation (or current continuity) on a coarse‐grained spatial scale. Quite intuitively, currents crossing cellular membranes become the extracellular currents.

Equation (17) expresses the relationship between extracellular currents and the CSD and does not require the introduction of an equivocal impressed current, which is sometimes invoked to explain the method of CSD analysis (Nicholson & Llinas, 1971; Hämäläinen *et al*., 1993; Nunez & Srinivasan, 2006). As discussed in the section ‘Fine‐grained description of electric currents in the extracellular space’, any current in brain tissue may arise due to electromigration, diffusion, advection and the displacement mechanisms, making any additional notion of a current superfluous. Here, we obviate the need for the impressed current because we explicitly perform the spatial averaging, which allows us to relate the extracellular and transmembrane currents.

In the presence of extracellular stimulation, the boundary of the extracellular space will also include electrode sites. Applying the same averaging in the presence of the electrode current results in a more general relationship:

(18) $$\nabla \cdot {\left\langle J^{\mathrm{tot}}\left(r\right)\right\rangle }_{e}=s^{\mathrm{mem}}\left(r\right)+s^{\mathrm{el}}\left(r\right),$$

where *s* ^el^(***r***) represents the additional electrode's current source. When approximating the electrode sites as points without spatial extent, after coarse‐graining we find $s^{\mathrm{el}}\left(r\right)=I_{k}w\left(r-r_{k}^{\mathrm{el}}\right)$, where *I*_*k*_ is the current leaving the electrode at the *k*‐th site located at $r_{k}^{\mathrm{el}}$.

In the section ‘Fine‐grained description of electric currents in the extracellular space’, we established that for fields of physiological origin, the advective and displacement currents in the extracellular space may be neglected, so that the total current ⟨ ***J*** ^tot^ ⟩_*e*_ = ⟨ ***J*** ^ohm^ + ***J*** ^dif^ ⟩_*e*_ may include only the Ohmic drift and the diffusion component. The extracellular electric field not only depends on the material properties of the extracellular space but also on the material properties of surrounding cells via the boundary conditions. As such, the electric field and the corresponding drift current in the extracellular space depend on the material properties of neural tissue (i.e., including both cellular and extracellular space). As we are interested in the coarse‐grained description, we are not concerned with the details of the electric field distribution in the extracellular space and the many intricate physical phenomena determining its dispersion properties (Foster & Schwan, 1995). Instead, we introduce the phenomenological relationship between the Fourier components of Ohmic current ${\left\langle J_{\omega }^{\mathrm{ohm}}\right\rangle }_{e}$ and the extracellular potential ⟨Φ_*ω*_ ⟩_*e*_ at the coarse‐grained scale:

(19) $${\left\langle J_{\omega }^{\mathrm{ohm}}\right\rangle }_{e}\overset{\mathrm{def}}{=}-{\overset{¯}{\sigma }}_{\omega }^{*}\nabla {\left\langle \Phi_{\omega }\right\rangle }_{e},$$

where ${\overset{¯}{\sigma }}_{\omega }^{*}={\overset{¯}{\sigma }}_{\omega }+i\omega {\overset{¯}{\varepsilon }}_{\omega }$ directly corresponds to the tissue conductivity, which may be seen by considering a four‐electrode system for measuring tissue impedance (Plonsey, 1969; Logothetis *et al*., 2007).

In such a configuration, the four electrodes are inserted in the extracellular space within the brain tissue and positioned along a straight line at equal distances *a*. The outer electrodes carry the applied current *I*_*ω*_ at the angular frequency *ω*, while the inner electrodes are used to measure the extracellular voltage Δ⟨Φ_*ω*_ ⟩_*e*_. When the electrode current source is much stronger than the CSD of membrane currents, Eq. (18) predicts the extracellular current to spread radially ${\left\langle J_{\omega }^{\mathrm{tot}}\right\rangle }_{e}=I_{\omega }/\left(4\pi {\left|r-r^{\mathrm{el}}\right|}^{2}\right)$ from the electrode for the locations deep within a volume of tissue. Considering the tissue with relatively uniform ionic concentrations, we may neglect the diffusion current as typically done in experiments for measuring tissue conductivity, so that ${\left\langle J_{\omega }^{\mathrm{tot}}\right\rangle }_{e}={\left\langle J_{\omega }^{\mathrm{ohm}}\right\rangle }_{e}$. Applying Eq. (19), we find the potential difference between the extracellular recording electrodes $\Delta {\left\langle \Phi_{\omega }\right\rangle }_{e}=I_{\omega }/\left(4\pi a{\overset{¯}{\sigma }}_{\omega }^{*}\right)$. This exact equation is used for calculating tissue conductivity from the measurements of extracellular voltage Δ⟨Φ_*ω*_ ⟩_*e*_ in a four‐electrode configuration experiment (Logothetis *et al*., 2007). Consequently, ${\overset{¯}{\sigma }}_{\omega }^{*}$ in Eq. (19) corresponds to the experimentally measured complex tissue conductivity.

Expressing Eq. (18) in the Fourier domain and utilizing Eq. (19) in place of the Ohmic current density we finally arrive at

(20) $$\nabla \cdot \left({\overset{¯}{\sigma }}_{\omega }^{*}\nabla {\left\langle \Phi_{\omega }\right\rangle }_{e}\right)=\nabla \cdot {\left\langle J_{\omega }^{\mathrm{dif}}\right\rangle }_{e}-s_{\omega }^{\mathrm{mem}}-s_{\omega }^{\mathrm{el}}.$$

This is the governing equation for the coarse‐grained extracellular potential ⟨Φ_*ω*_ ⟩_*e*_. It states that, in general, the extracellular potential is determined by the CSD of tramsmembrane currents $s_{\omega }^{\mathrm{mem}}$, the divergence of the diffusive current $-\nabla \cdot {\left\langle J_{\omega }^{\mathrm{dif}}\right\rangle }_{e}$ as well as by the stimulating electrode source. A unique solution to Eq. (20) within a volume of brain tissue may be found for a given distribution of CSD, diffusion current and electrode sources when supplemented by the boundary conditions for the potential or its normal derivative on the surface enclosing the volume.

In the absence of the electrode current and if the frequency dependence of the tissue conductivity in the physiological range may be neglected (Logothetis *et al*., 2007; Miceli *et al*., 2017), Eq. (20) simplifies to:

(21) $$\nabla \cdot \left(\overset{¯}{\sigma }\nabla {\left\langle \Phi \right\rangle }_{e}\right)=\nabla \cdot {\left\langle J^{\mathrm{dif}}\right\rangle }_{e}-s^{\mathrm{mem}},$$

where now all terms have identical temporal dynamics. Finally, when conductivity is uniform and isotropic, and the gradients of diffusion currents are negligible in comparison to the CSD, Eq. (21) further simplifies to

(22) $$\overset{¯}{\sigma }\nabla^{2}{\left\langle \Phi \right\rangle }_{e}=-s^{\mathrm{mem}},$$

which recovers the result of the original theory of CSD analysis, except that here the Poisson equation is explicitly formulated for the coarse‐grained extracellular potential.

### Effects of extracellular diffusion on the LFP recordings

**Figure 2:**
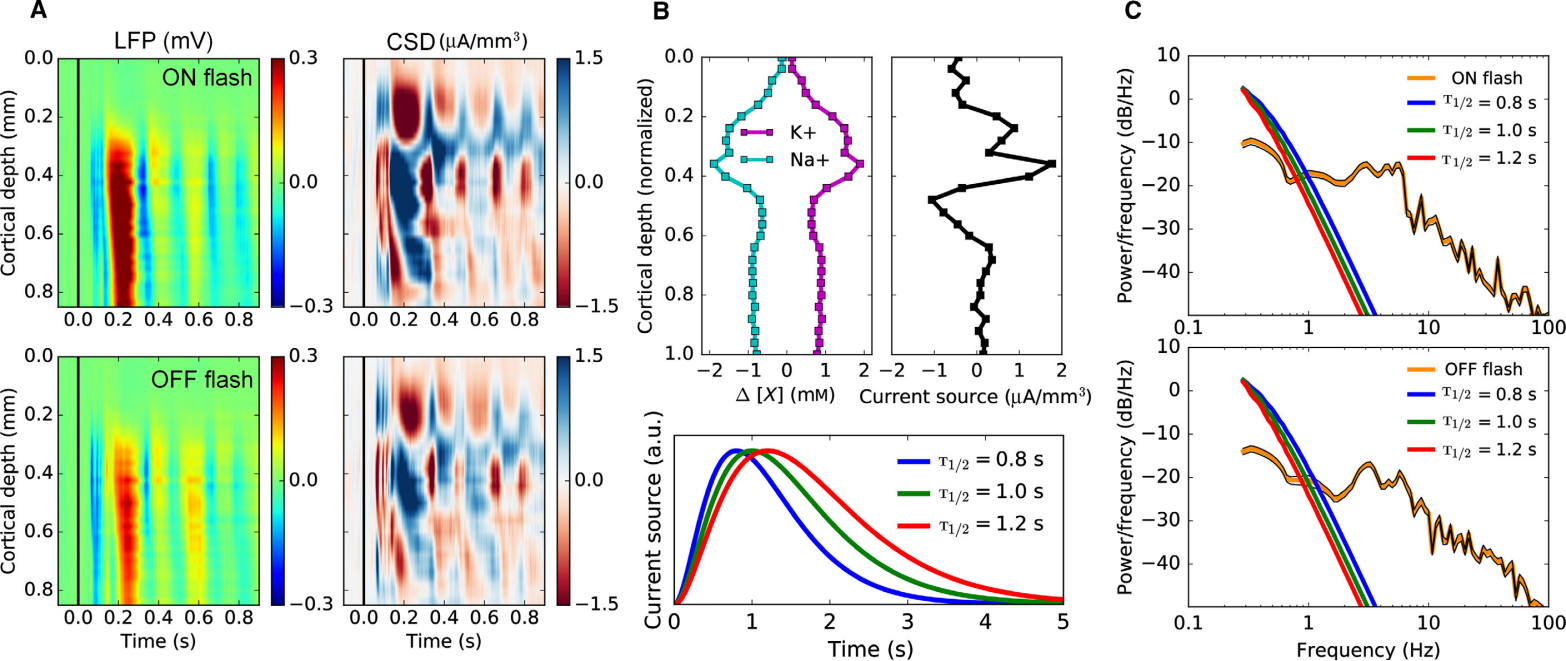
Estimation of the contribution of extracellular diffusion toward the LFP recordings. (A) Trial‐averaged LFP recordings (left) and the corresponding CSD estimates (right) from the mouse visual cortex in response to the presentation of the full‐field 50 ms flash: ‘ON flash’ (top) and ‘OFF flash’ (bottom). Black vertical line indicates the stimulus onset. (B) The experimentally recorded spatial profile of the extracellular [K+] in cat visual cortex in response to the electrical stimulation of the thalamus (Modified with permission from Cordingley & Somjen (1978), Fig. 5) and the corresponding assumed [Na+] profile (top left). The estimated spatial profile of the apparent CSD resulting from diffusion (top right). The modeled [K+] transients corresponding to the experimentally recorded half‐decay times (Cordingley & Somjen, 1978) for the post‐stimulus clearance (bottom). (C) Comparison of the power spectral density of the estimated apparent CSD resulting from diffusion (B) for the three modeled half‐decay times (red, green and blue lines) and of the CSD from (A) averaged over the cortical depths (mean: orange line, +/‐SEM: black lines) for the ‘ON flash’ (top) and ‘OFF flash’ (bottom) stimulus conditions.

Typically, the diffusion component of the extracellular current is tacitly neglected in CSD analysis. Here, we give a crude estimate of the impact that diffusive currents can have on the LFP under conditions where extracellular concentration gradients become relatively large. As obtaining high‐resolution data of both extracellular potentials and ion concentrations simultaneously from the same volume is not feasible, we based our estimate on comparing two independent experiments: (1) We recorded the extracellular potential *in vivo* from the mouse primary visual cortex with a high‐density multi‐electrode array (inter‐electrode spacing ~20 μm) in response to the repeated presentations of visual stimuli (see Materials and methods: Electrophysiological recordings). For these data, the CSD was estimated (see Materials and methods: Estimation of the CSD) from the trial‐averaged LFP responses based on Eq. (22), that is, under the assumption that transmembrane currents are the sole contributors to the extracellular potential (Fig. 2 A). (2) We used previously published data for the extracellular [K+] transients in the mammalian cortex arising in response to the electrical stimulation of the thalamus (Cordingley & Somjen, 1978). Accounting for electroneutrality in the extracellular space, we also assumed, as a first approximation, that increases in the extracellular [K+] are compensated by the equal decreases in the extracellular [Na+], which is in qualitative agreement with experimental findings (Dietzel *et al*., 1982). Using these data, we estimated the divergence of the diffusive current (i.e., the apparent CSD resulting from diffusion) $-\nabla \cdot {\left\langle J^{\mathrm{dif}}\right\rangle }_{e}$ (Fig. 2B).

Finally, in Fig. 2C, we compared the spectral power of the estimated CSD in (A) to the apparent CSD obtained from the diffusive process in (B). We observe that the divergence of the diffusive current has the highest power at frequencies ≲1 Hz, but rapidly attenuates to become negligible at higher frequencies. Hence, under the presence of relatively large extracellular concentration gradients, the low frequency part of the LFP is likely to be influenced by diffusive currents, which cannot be neglected when estimating the CSD. The sharp drop off in the power spectrum of the apparent CSD due to diffusion is determined by the relatively slow temporal dynamics of the extracellular ionic concentration build‐up and clearance observed for different experimental conditions (Cordingley & Somjen, 1978; Connors *et al*., 1979). As such, we expect this result to be more generally applicable to a wide range of experimental conditions.

## Discussion

The theory developed in this paper is motivated by the limiting and implicit assumption of the theory of CSD analysis and the ensuing challenges to its validity. The original theory of CSD analysis (Nicholson, 1973) was developed in the limit of a quasi‐stationary approximation of Maxwell's equations, assuming a frequency‐independent Ohmic extracellular material and neglecting the possible diffusive, advective and displacement currents in the extracellular space. Here, starting with the macroscopic Maxwell's equations we justify the application of the electro‐quasistatic approximation to describe fields in the brain of physiological origin, which neglects only the magnetic induction but accounts for the displacement current. Then, we describe a coarse‐graining procedure by which the field variables are averaged separately over cellular and the extracellular spaces. This naturally leads to the notion of the CSD, which describes the membrane current (both ionic and synaptic) at the coarse‐grained scale suitable for the analysis of sparsely sampled experimental recordings. Accounting for all possible mechanisms of charge transfer we show that, in general, both the CSD as well as gradients of extracellular diffusion currents determine the coarse‐grained extracellular potential, which satisfies the Poisson equation. The advective and displacement current in the extracellular place may be neglected for frequencies of physiological origin. On the other hand, the displacement current in the cellular space is included as a part of CSD.

### The practice of CSD analysis

Depending on the assumptions about properties and processes occurring in brain tissue, either Eq. (20), (21) or (22) could be used to estimate the CSD of membrane currents from the recorded extracellular potential. When neither the frequency dependence of conductivity nor the diffusion currents can be neglected, Eq. (20) must be used to estimate the CSD of membrane currents separately at each temporal frequency. Only if the frequency dependence of extracellular conductivity is negligible can Eq. (21) be used. In the simplest situation of constant conductivity and no diffusion currents, Eq. (22) may be used instead. Conversely, when diffusion currents cannot be neglected, one must estimate them independently and, per Eq. (21), subtract them from the Laplacian to arrive at the CSD of membrane currents.

When applied to experimental data, one must overcome a number of issues (Freeman & Nicholson, 1975): 1) electrical recordings are rarely available to evaluate the Laplacian across all three spatial dimensions, necessitating additional assumptions regarding the source distribution; 2) noise in the experimental data is significantly amplified by spatial differentiation, requiring the use of noise regularization strategies. These challenges motivated the development of several methods and respective software tools for estimating the CSD (Pettersen *et al*., 2006; Łęski *et al*., 2007, 2011; Potworowski *et al*., 2012) which have been successfully applied to analyze experimental recordings.

Both the coarse‐grained extracellular potential and the CSD in the governing equation utilize the same averaging kernel and correspondingly have the same spatial resolution. Therefore, the spatial resolution of the estimated CSD is determined by the spatial resolution of the data, that is, the inter‐channel spacing on the electrode shank. Whether the selection of a particular spacing is sufficient for a particular application depends on the spatial frequencies of interest, which need to be resolved. The spatial localization of the underlying neuronal currents is in part determined by the temporal dynamics of the electrical activity because of the frequency dependence of the membrane impedance. For instance, the membrane length constant in passive dendrites is inversely proportional to the temporal frequency (Koch, 2004) such that membrane currents at higher temporal frequencies decay faster along the cable (i.e., more localized) than those at lower temporal frequencies (Lindén *et al*., 2010; Anastassiou *et al*., 2015). Correspondingly, as manufacturing technology continues to advance toward increasing channel density, more localized current sources can be resolved, which are typically characterized by faster temporal dynamics.

### Difference between the fine‐grained and coarse‐grained descriptions of extracellular potential

The limited spatial resolution of extracellular recordings dictates the need for a coarse‐grained description of membrane current sources and the extracellular potential in brain tissue. Here, we developed such a description by performing spatial averaging of currents while distinguishing between the cellular and extracellular spaces. Applying the mathematical identity developed in Appendix A, the coarse‐grained currents within the cellular space were expressed via a volume density of transmembrane currents, that is, the CSD. Consequently, we find that generally the Poisson equation describing the coarse‐grained extracellular potential, Eq. (21), includes both the CSD of transmembrane currents and the divergence of the diffusion currents as sources on the right‐hand side. In contrast, Eq. (10) describes the fine‐grained extracellular potential and may only include the divergence of diffusion currents as a source on the right‐hand side. The difference between the two descriptions lies in the way they account for the boundary conditions, that is, the transmembrane currents. The solution of Eq. (10) for the extracellular potential is sought within the narrow confines of extracellular space and the effects of the transmembrane currents are included through the boundary conditions. On the other hand, the solution of Eq. (21) is sought within the tissue and the contribution of the membrane currents is included in the CSD. Furthermore, Eq. (10) includes the conductivity of extracellular space while Eq. (21) includes tissue conductivity.

### Diffusion currents in the extracellular space

Several pathological conditions, such as hypoxia, anoxia, ischemia and spreading depression are associated with significant ion concentration changes in the extracellular space (Syková & Nicholson, 2008). Also during non‐pathological conditions, neural signaling may cause local ion concentration changes. For example, [K+]_e_ elevations in the cat striate cortex in response to bright bars moving across the receptive field amount to ~0.1 mM (Connors *et al*., 1979), whereas strong repeated cortical stimulation may locally elevate [K+]_e_ up to 10 mM (Pumain & Heinemann, 1985).

Extracellular ion concentration changes are typically inhomogeneous across the cortical depth (Cordingley & Somjen, 1978; Nicholson *et al*., 1978; Pumain & Heinemann, 1985). The presence of ionic concentration gradients results in ionic diffusion, which in turn gives rise to electrical current in the extracellular space. As for temporal dynamics, extracellular [K+] builds‐up and clears with a time constant ≳1 s (Cordingley & Somjen, 1978; Connors *et al*., 1979). Diffusion currents due to extracellular concentration gradients are thus likely to change at a slow time scale of seconds and correspondingly are expected to contribute only to the low frequency components of the LFP. In the present application, we found that extracellular diffusion may be of importance for determining the LFP at frequencies ≲1 Hz (see section ‘Effects of extracellular diffusion on the LFP recordings’) for physiological conditions accompanied by strong (≳1 mM) changes in the extracellular ionic concentrations. Similar results were found in a previous computational study, where diffusive currents were found to influence LFP frequency components up to a few Hz in the case of large extracellular concentration gradients (Halnes *et al*., 2016).

How do these findings change our interpretation of the depth LFP with regard to ongoing activity? Being a low‐frequency effect, extracellular diffusion is unlikely to play a role in oscillations such as theta (2–12 Hz), beta (12–30 Hz), gamma (30–80 Hz), etc. On the other hand, several slower oscillatory patterns exist with their main frequency component being below 1 Hz such as slow neocortical rhythms and delta waves (Gloor *et al*., 1977; Buzsáki *et al*., 1988; Steriade *et al*., 1993). These patterns have been shown to play key role in neural functioning and coordination. For example, slow neocortical activity with its accompanying UP–DOWN states critically contributes to the temporal organization of other cortical patterns, such as sleep spindles, gamma oscillations and K‐complexes (Achermann & Borbely, 1997; Steriade & Amzica, 1998; Mölle *et al*., 2002; Mukovski *et al*., 2007) as well as hippocampal sharp wave ripples (Sirota *et al*., 2003; Sirota & Buzsáki, 2005). Hitherto, the source of the extracellular signal associated with slow neocortical oscillations has been chiefly ascribed to intracellular UP–DOWN dynamics and rhythmic polarization (extending 10–20 mV) of cortical neurons. Yet, a progressive decrease in extracellular calcium concentration by approximately 20% has also been measured during UP states. It has been hypothesized that such Ca concentration changes can lead to decrease in neurotransmitter release probability and, eventually, promote the subsequent DOWN state (Massimini & Amzica, 2001). Yet, our work suggests an additional role of such Ca concentration change, as they can affect the CSD estimates. Our study suggests an alternative interpretation of the signals associated with slow neocortical activity where a significant part of the signal below 1 Hz may be contributed by ionic diffusion with the rest of it associated with neural membrane polarization.

Here, we propose a way to account for the effects of extracellular ionic diffusion on the extracellular potential. The theory shows that the extracellular potential at the coarse‐grained scale is governed by Poisson's equation (Eq. (21)) with the source term generally including the membrane currents and contributions from extracellular diffusion. When the diffusion current gradients can be neglected, the extracellular potential is still determined by Poisson's equation in accordance with the original theory of CSD analysis (Nicholson, 1973). As such, our theory is the generalization of the original theory of CSD analysis.

Nevertheless, our findings contrast with the theory of Bédard & Destexhe (2011), who predicted that the coarse‐grained extracellular potential is governed by Poisson's equation only when the diffusion effects are included (see Eq. (11) in Bédard & Destexhe (2011)), otherwise the extracellular potential is governed by the Laplace equation. Consequently, it would follow that the CSD estimated by computing a Laplacian of the extracellularly recorded potential must be interpreted as the divergence of the extracellular diffusion currents. Naturally, such a finding would suggest an essential role for extracellular ionic diffusion currents in determining the extracellular potential. However, we believe that this conclusion is erroneous. Equation (11) in Bédard & Destexhe (2011) has the same physical meaning as Eq. (10) in this paper. As discussed in the section ‘Fine‐grained description of electric currents in the extracellular space’, the application of Eq. (10) to describe the extracellular potential requires solving it within the narrow confines of the extracellular space and consequently requires specifying the boundary conditions along the cellular membrane. The need for the explicit boundary condition along cellular membrane makes Eq. (10) and correspondingly Eq. (11) in Bédard & Destexhe (2011) unsuitable for the analysis of extracellular recordings at the coarse‐grained scale. In contrast, here we developed the formalism for describing the extracellular potential at the coarse‐grained scale within a tissue space by incorporating the membrane currents into the CSD term in the Poisson equation that makes it suitable for the analysis of LFP recordings.

## Conflicts of interest

The authors declare no conflicts of interest, financial or otherwise.

## Data accessibility

Data from electrophysiological recordings used in this study are archived at: https://doi.org/10.6084/m9.figshare.4780321

## Author contributions

SLG, GH, and GTE developed the theory. DD conducted in vivo experiments and SLG analyzed experimental data. SLG, GH, GTE, MJH, CK and CAA wrote the paper.

**Abbreviations**
LFP: local field potential
CSD: current‐source density

## Appendix A Coarse‐graining

Here, we develop the expression for the divergence of a vector field averaged over a volume bound by a generally convoluted and tortuous surface (or a collection of surfaces) such as neuronal membranes. Subsequently, we will use the derived expression to formally define the transmembrane CSD.

Let us consider an arbitrary differentiable vector field ***F***(***r***) and define the averaged field over the volume *V*_*c*_:

(23) $${\left\langle F\left(r\right)\right\rangle }_{c}\overset{\mathrm{def}}{=}\int_{V_{c}}dv^{\prime }F\left(r^{\prime }\right)w\left(r-r^{\prime }\right)$$

bounded by an arbitrarily torturous surface *S*_*c*_ such as cellular membrane. To simplify the notation, we will use Einstein's convention for index summation such that the divergence $\nabla \cdot F\equiv \nabla_{i}F_{i}$, where $\nabla_{i}=\frac{\partial }{\partial r_{i}}$ is the derivative with respect to the *r*_*i*_ component of the position ***r*** = (*r*_1_, *r*_2_, *r*_3_). Taking the divergence of the vector field in Eq. (23) we find:

(24) $$\nabla_{i}{\left\langle F_{i}\left(r\right)\right\rangle }_{c}=\int_{V_{c}}dv^{\prime }F_{i}\left(r^{\prime }\right)\nabla_{i}w\left(r-r^{\prime }\right),$$

where the derivative may be moved inside the integration because differentiation and averaging act on different variables. Noting that $\nabla_{i}w\left(r-r^{\prime }\right)=-\nabla_{i}^{\prime }w\left(r-r^{\prime }\right)$ we have

(25) $$\nabla_{i}{\left\langle F_{i}\left(r\right)\right\rangle }_{c}=-\int_{V_{c}}dv^{\prime }F_{i}\left(r^{\prime }\right)\nabla_{i}^{\prime }w\left(r-r^{\prime }\right),$$

where now the differentiation and integration are performed with respect to the primed coordinates ***r′***. Since the integrand on the right‐hand side may be expressed as:

(26) $$-F_{i}\left(r^{\prime }\right)\nabla_{i}^{\prime }w\left(r-r^{\prime }\right)=-\nabla_{i}^{\prime }\left(w\left(r-r^{\prime }\right)F_{i}\left(r^{\prime }\right)\right)+w\left(r-r^{\prime }\right)\nabla_{i}^{\prime }F_{i}\left(r^{\prime }\right),$$

we find that

(27) $$\nabla_{i}{\left\langle F_{i}\left(r\right)\right\rangle }_{c}=-\int_{V_{c}}dv^{\prime }\nabla_{i}^{\prime }\left(w(r-r^{\prime }\right)F_{i}\left(r^{\prime }\right))+\int_{V_{c}}dv^{\prime }w\left(r-r^{\prime }\right)\nabla_{i}^{\prime }F_{i}\left(r^{\prime }\right).$$

Applying Gauss’ theorem to the first term and the definition of the averaged field, Eq. (22), to the second term on the right‐hand side, we arrive at the identity expressed in vector notation:

(28) $$\nabla \cdot {\left\langle F\left(r\right)\right\rangle }_{c}=-\int_{S_{c}}da^{\prime }\cdot F\left(r^{\prime }\right)w\left(r-r^{\prime }\right)+{\left\langle \nabla \cdot F\left(r\right)\right\rangle }_{c},$$

where we put $da^{\prime }\overset{\mathrm{def}}{=}nda^{\prime }$ with ***n*** being a unit surface normal. The appearance of the surface term on the right‐hand side indicates that, in general, the averaging and differentiation do not commute!

Commonly, averaging is performed over the entire space such that the bounding surface *S*_*c*_ is infinitely removed from the region of interest in space. As the averaging kernel is non‐zero only within a neighborhood $\left|r-r{}^{\prime }\right|<R$ around each field point ***r***, the surface term vanishes, resulting in commutativity between averaging and differentiation:

(29) ∇·⟨F(r)⟩=⟨∇·F(r)⟩.

Conversely, when the bounding surface crosses the neighborhood |***r*** − ***r***′| < *R*, we must use a general identity, Eq. (28). In particular, this applies when averaging fields over the cellular space, because there the averaging volume includes many bounding surface (i.e., surface membranes) as illustrated in Fig. 1 B.

The surface term in Eq. (28) is essential when applied to the total current density ***J*** ^tot^(***r***). As the total current density is solenoidal, $\nabla \cdot J^{\mathrm{tot}}=0$, we find:

(30) $$\nabla \cdot {\left\langle J^{\mathrm{tot}}\left(r\right)\right\rangle }_{c}=-\int_{S_{c}}da^{\prime }J_{m}\left(r^{\prime }\right)w\left(r-r^{\prime }\right),$$

where we defined the boundary current density $J_{m}\overset{\mathrm{def}}{=}n\cdot J^{\mathrm{tot}}$. Equation (30) states that current density averaged over a volume *V*_*c*_ equals a negative sum of the currents—weighted by the averaging kernel—over the surface *S*_*c*_ bounding the volume. If *S*_*c*_ represents a cellular membrane, then Eq. (30) states that the current density averaged over the cellular volume $\nabla \cdot {\left\langle J\left(r\right)\right\rangle }_{c}$ equals the negative weighted sum of the transmembrane current density *J*_*m*_(***r***).

## Appendix B On the use of the electroneutrality assumption in the presence of diffusion fluxes

In the section ‘Fine‐grained description of electric currents in the extracellular space’ we used the current continuity Eq. (6) to derive Eq. (10) describing the extracellular potential in the presence of diffusion. On the other hand, applying the electro‐quasistatic approximation to the Gauss's law we find:

(31) ∇·(ε∇Φ)=−ρ,

which must also be satisfied by the extracellular potential. For physiological frequencies *ωτ*_*e*_ ≪ 1 and consequently from Eq. (9), we find the charge density:

(32) $$\rho =\tau_{e}F\sum_{i}z_{i}\nabla \cdot \left(D_{i}\nabla c_{i}\right),$$

where *ρ* = *ρ* ^*f*^ + *ρ* ^*m*^ includes the contributions of the fixed charges *ρ* ^*f*^ on the extracellular matrix and mobile charges $\rho^{m}=F\sum_{i}z_{i}c_{i},$ in the interstitial fluid. Assuming, for simplicity, the homogeneous electrical properties and substituting the expression for the charge density in Eq. (32) into Eq. (31) we find:

(33) $$\sigma \nabla^{2}\Phi =-F\sum_{i}z_{i}\nabla \cdot \left(D_{i}\nabla c_{i}\right),$$

which is the special case of Eq. (10) with constant conductivity. Thus, both Gauss's law and the charge continuity result in consistent equations for describing the extracellular potential.

On the other hand, if exact electroneutrality were to be assumed, that is, *ρ* = 0, then from Eq. (31) we would end up with the Laplace equation ∇^2^ *Φ* = 0, leading to an inconsistency with Eq. (33) and, correspondingly, a more general Eq. (10). Thus, strict electroneutrality cannot be assumed for the purposes of describing the electric field in the presence of diffusion fluxes.
